# Challenges to establish the diagnosis of aspergillosis in non-laboratory animals: looking for alternatives in veterinary medicine and demonstration of feasibility through two concrete examples in penguins and dolphins

**DOI:** 10.3389/fcimb.2022.757200

**Published:** 2022-07-19

**Authors:** Guillaume Desoubeaux, Carolyn Cray, Adélaïde Chesnay

**Affiliations:** ^1^ Parasitologie – Mycologie – Médecine tropicale, Hôpital Bretonneau, CHRU Tours, Tours, France; ^2^ Centre d’étude des pathologies respiratoires – Inserm U1100, faculté de Médecine, Université de Tours, Tours, France; ^3^ University of Miami, Comparative Pathology, Miller School of Medicine, Miami, FL, United States

**Keywords:** Sphenisciformes, *Spheniscus*, *Tursiops*, western blot, cetaceans, mass spectrometry, iTRAQ (Isobaric tagged for relative and absolute quantitation), protein electrophoresis

## Abstract

Aspergillosis remains difficult to diagnose in animals. Laboratory-based assays are far less developed than those for human medicine, and only few studies have been completed to validate their utility in routine veterinary diagnostics. To overcome the current limitations, veterinarians and researchers have to propose alternative methods including extrapolating from human diagnostic tools and using innovative technology. In the present overview, two specific examples were complementarily addressed in penguins and dolphins to illustrate how is challenging the diagnosis of aspergillosis in animals. Specific focus will be made on the novel application of simple testing in blood based on serological assays or protein electrophoresis and on the new information garnered from metabolomics/proteomics to discover potential new biomarkers. In conclusion, while the diagnostic approach of aspergillosis in veterinary medicine cannot be directly taken from options developed for human medicine, it can certainly serve as inspiration.

## Introduction

Aspergillosis is a fungal airborne disease caused by ubiquitous molds belonging to the *Aspergillus* genus, and primarily by *Aspergillus fumigatus* species (Desoubeaux et al., 2014). In animals, aspergillosis can infect a wide range of species from invertebrates, such as corals, to higher vertebrates (Seyedmousavi et al., 2015). In the latter, the course of the disease and the clinical signs can vary greatly. For instance in penguins, aspergillosis is mostly observed in individuals managed under human care and is represented by the subacute development of granuloma and plaques at the surface of lungs and air sacs (Desoubeaux et al., 2018; Cateau et al., 2022). In contrast in cetaceans, aspergillosis course is based on a chronic invasive process of lungs, and then other organs like brain, which is generally indicative of another disease and/or (sub-)acute physiologic stress (Bunskoek et al., 2017; Desoubeaux et al., 2018), but it is rarely associated with severe immunosuppression and profound neutropenia (Seyedmousavi et al., 2015)..

For veterinarians and all staff that takes care of animals, the diagnosis of aspergillosis is often quite challenging because laboratories tools are neither numerous nor accurate enough (Cray et al., 2009; Desoubeaux et al., 2018; Elad and Segal, 2018; Tell et al., 2019), and medical imaging is not readily available in every facility (Jones and Orosz, 2000). Furthermore, there is no approved classification for helping to rank the cases according to the level of evidence, as is found in human medicine (Donnelly et al., 2020).

In order to more accurately address the diagnosis of aspergillosis in animals, a novel approach can consist in trying to extrapolate new tools initially-intended for human medicine to veterinary one. Another possibility is identifying high-put screening potential biomarkers by the means of innovative technology such as metabolomics or proteomics. Thus, in the present article primarily intended to veterinarians and staff of animal diagnostic laboratory, we will specifically discuss two complementary examples in penguins and dolphins to illustrate studies of aspergillosis in animals and diagnostic options which have been defined in this research.

## What are the options to achieve a more accurate diagnosis in penguins?

A large number of avian species can be infected with *Aspergillus* (Seyedmousavi et al., 2015). Penguins are especially at risk (Desoubeaux et al., 2018)3 Several reasons have been raised for explaining this finding. First, most species belonging to Spheniscidae family that have burrowing behavior, *e.g*. Magellanic (*Spheniscus magellanicus*), Humboldt (*Spheniscus humboldti*) or African penguins (*Spheniscus demersus*), so that the birds are potentially exposed to fungal spore clouds when disturbing soil. Secondly, captive conditions in zoological parks or aquaria can enhance the presence of stress related factors which may predispose penguins to aspergillosis: for example, massive afflux of visitors, long transfers between two facilities, dirty habitats with dampness, and bad ventilation or excess of ammonium derivatives (Miller and Fowler, 2014; Terio et al., 2018). Moreover, aspergillosis seems more commonly diagnosed in juveniles – potentially more fragile birds – than adults (Xavier et al., 2007; Terio et al., 2018; Cateau et al., 2022). Altogether, its relative prevalence can reach 20-27% in some penguin colonies under managed care (Filho RP da et al., 2015; Krol et al., 2020), and its incidence was recently measured at ~3.4% case-years in a French zoological park (Cateau et al., 2022). However, one should be aware that great discrepancies can be observed between centers depending on the occurrence variations of co-morbidities and the availability of diagnostic means of diagnosis (e.g. access to medical imaging).

The clinical course of aspergillosis is mostly subacute or chronic in birds (Seyedmousavi et al., 2015). It can lead to weight loss and lethargy, open mouth-breathing, wheezing, coughing, altered vocalization and dyspnea (Miller and Fowler, 2014), and eventually death in up to 50% infected penguins (Filho RP da et al., 2015). *Antemortem* diagnostics can involve a host of ancillary testing including routine hematology, biochemistry, and radiography among others many of which can only provide supportive or suggestive results to help form the diagnosis (Jones and Orosz, 2000). The curative treatment is based on azole drugs, like voriconazole tablets hidden in the food or itraconazole suspension administrated by pipette directly into the esophagus. Mass antifungal prophylaxis is not practiced in penguins and usually restricted only to individuals that are fragile or at risk (Xavier et al., 2007; Miller and Fowler, 2014).

Necropsy can offer the possibility to observe nodules in the lung parenchyma. In addition, whitish plaques are sometimes visible in the air sacs and at the inner surface of the trachea providing conclusive confirmation of the fungal etiology (Desoubeaux et al., 2014). The direct examination through histopathology or cytology can reach up 90% sensitivity (personal data; under submission). In contrast, *ante mortem* diagnostics usually have poor performance. First, biopsy sampling can be difficult to perform in ill or debilitated subjects. Colony-forming unit (CFU) counting based on *in vitro* mycological culture is not reliable enough to estimate the actual *Aspergillus* burden, because it does not reflect the total amount of viable, dormant and dead fungus within the tissue or the fluid that is investigated (Desoubeaux et al., 2014; Melloul et al., 2014). Since the penguin respiratory system may be colonized by *Aspergillus* spp., it may be difficult to distinguish between true infection and simple colonization on the basis of the culture alone (Desoubeaux et al., 2014). Detection of galactomannan antigen in plasma is not reliable in some birds and is frequently found falsely negative or only slightly positive in infected penguins (Cray et al., 2009). For example, Desoubeaux et al. reported no significant difference in mean galactomannan index of 0.6, 0.5 and 0.2 in 47 *Aspergillus*-diseased African penguins, 29 control penguins with miscellaneous inflammatory conditions, and 96 clinically-normal penguins, respectively (*P*=0.14) (Desoubeaux et al., 2018). Some lateral flow devices with murine JF5 and MAb476 antibodies were developed to detect *Aspergillus* antigens in human blood, relying on the same technical principle as for a pregnancy test in urine (Savelieff et al., 2018). These devices were tested in birds, mostly penguins, but with poor results to date (personal data unpublished).

The (Desoubeaux et al., 2014; Seyedmousavi et al., 2015; Cateau et al., 2022)-β-D-glucan is a cell wall pan-fungal component with a high negative predictive value (Donnelly et al., 2020), but with no specificity to *Aspergillus* genus. Highly variable levels of this marker were reported in experimentally and naturally infected birds, as well as control birds, negating then its potential ready application to avian medicine (Burco et al., 2012). Its sensitivity and specificity were assessed at 60.0 and 92.7% with an elevated cutoff established at 461 pg/dL (*vs.* 80 in human medicine). Measurement of anti-*Aspergillus* antibody by the means of ELISA kits based on a crude antigen preparation has been reported to be consistently positive in penguin blood regardless of clinical status (Cray et al., 2009). In the aforementioned study, the mean indices of antibody were 1.8, 1.7 and 1.7 for the infected population, the inflammatory controls, and the healthy subjects (according to a positive cutoff established at 1.4 index), respectively (Desoubeaux et al., 2018). Recently listed among the diagnostic options acknowledged for the use in humans (Donnelly et al., 2020), *Aspergillus* qPCR has been rarely reported in birds (Melloul et al., 2014). Usually, it targets the ribosomal RNA subunits, like the 28S subunit (Chauvin et al., 2019). In a recent study performed in a large French zoological park, its sensitivity and specificity performance were evaluated at ≈84% and 100% in lung biopsies obtained from deceased individuals belonging to a colony of ≈130 Humboldt penguins (Cateau et al., 2022).

**Figure 1:**
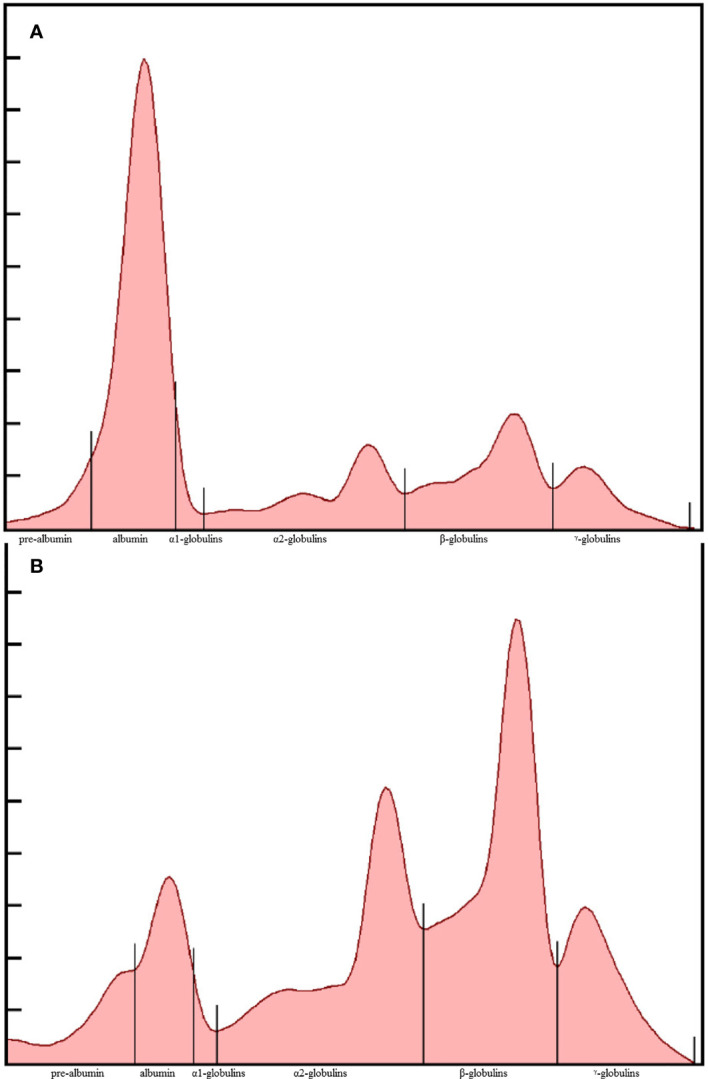
Representative plasma protein electrophoretograms of a clinically-normal **(A)** and *Aspergillus-*diseased African penguin (*Spheniscus demersus*) **(B)**. The fractions, from left to right, are prealbumin, albumin, α1, α2, β, and γ - globulins.

**Figure 2:**
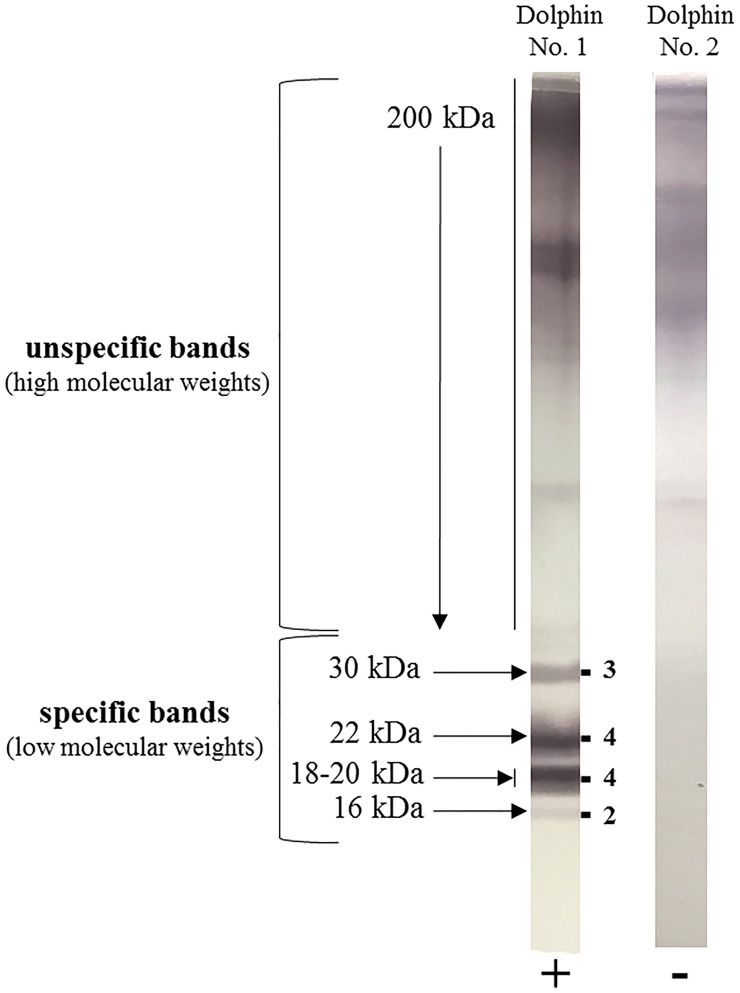
Example of two blood samples from common bottlenose dolphins (*Tursiops truncatus*) tested for anti-*Aspergillus* antibody by the *Aspergillus* Western blot IgG^®^ kit (LDBio Diagnostics, Lyon, France). Dolphin N°1 was found positive (+) with a global Western blot score of 13/16, as indicated on the right side of the immunoblot strip by the sum of the respective band intensities observed at 30, 22, 18-20 and 16 kDa. Dolphin N°2 was found negative (-).

As simple and low-cost alternative, some authors suggested to focus on plasma protein electrophoresis (EPH) to address the diagnosis of aspergillosis (Cray et al., 2009). Valid quantitation of certain protein fractions through EPH was demonstrated to provide a reflection of acute phase responses (Cray et al., 2011): a decreased albumin was often observed in tandem with the increase of four globulin fractions, including α1-, α2-, β- and γ-globulins (Kaneko, 2008) (**Figure 1**, **2**). In another study, a moderate decrease in percent albumin was supportive of its designation as a negative acute phase protein and showed a strong negative predictive value for assessing survival in gentoo penguins (*Pygoscelis papua*) (Naylor et al., 2017). The clinical significance of prealbumin changes in birds has not been clearly defined, but a decrease was observed in experimentally-infected falcons (Kummrow et al., 2012; Fischer et al., 2014), and in naturally-infected African penguins (0.32 *vs*. 0.39g/dL in healthy controls; *P*=0.006) (Desoubeaux et al., 2018). Elevation of haptoglobin is proposed to be related to the approximate +0.33 g/dL change of α2-globulin fraction that was observed in some recent studies (Desoubeaux et al., 2018). Greater levels of γ-globulins are consistent with the stimulation of humoral immunity, and two fold increased *vs.* control animals were observed (Desoubeaux et al., 2018). Overall, the presence of these EPH based abnormalities is sufficient to initiate preemptive antifungal treatment. Semi-automated methods of protein fractionation have been commercialized for veterinary purposes over the last 20 years (Tatum et al., 2000; Kaneko, 2008; Cray et al., 2009; Cray et al., 2011).

Based on laboratory animal models, several studies suggest that the detection of gliotoxin could have a strong potential as diagnostic signature of aspergillosis (Lewis et al., 2005; Cerqueira et al., 2014; Sugui et al., 2017). Gliotoxin is produced by *Aspergillus* during its hyphal growth and is the most abundant mycotoxin playing the role of key-virulence factor that results in far-reaching immune suppression of the host (Kwon-Chung and Sugui, 2009; Arias et al., 2018). Through measurement with high-performance liquid chromatography-tandem mass spectrometry (HPLC-MS/MS) methodology, we recently tested the relevancy of gliotoxin detection for the diagnosis of aspergillosis in blood samples drawn from African, Humboldt, gentoo and Magellanic penguins among other avian species (Reidy et al., 2022). In all, gliotoxin was detected in almost 74% of the clinical samples obtained from birds with proven or probable aspergillosis, but was not detected in samples from clinically-normal penguins. A poor prognostic was associated to repeated measures from birds undergoing unsuccessful treatment. Gliotoxin positive rates were higher in confirmed rather than probable cases.

Recently, proteomic and metabolomic studies provided new opportunities of exploration for discovering potential biomarkers of infection including aspergillosis (Desoubeaux et al., 2014). For example, a significant increase of some ketone bodies, lipoprotein, and fatty acids, including 3-hydroxybutyrate, was firstly described by metabolomics in the blood of infected falcons (Pappalardo et al., 2014). Elevation of 3-hydroxybutyrate in plasma was subsequently confirmed in a cohort of 47 infected African penguins *vs.* 115 controls (Desoubeaux et al., 2018). Notably, it was associated with the increase of the absolute concentrations of β-globulins and α2-globulins. Using these measures in tandem resulted in high specificity (>90%) and high negative predictive value (≥80%), thus suggesting that basic EPH testing in combination with 3-hydroxybutyrate can provide reliable evidence for the absence of aspergillosis diagnosis, when all are negative. In the same study, when 3-hydroxybutyrate concentration was elevated > 1.9 mmol/L, the clinical prognosis was quite poor. In contrast, the levels returned to normal when penguins recovered. Through its great sensitivity, proteomics may contribute to the discovery of new markers of aspergillosis (Desoubeaux et al., 2014). Interestingly, a second example of exploratory study based on proteomics evidenced several significant changes in protein representation in infected penguins. For instance, F-box/LRR-repeat protein 4, THAP domain-containing protein 1, histidine-tRNA cytoplasmic ligase and AIM1 (absent in melanoma-1) protein were found 4.4-, 2.5-, 2.5-, 2.2-fold overexpressed in the blood of diseased subjects *vs.* non-*Aspergillus* inflammatory controls (Desoubeaux et al., 2018). Globally, it is noteworthy to report that three protein pathways were significantly enriched during aspergillosis processes: cadherin, Wnt and FGF signaling pathways. Cadherin pathway is involved in cell adhesion by forming *adherens* junctions to bind cells within tissues together (Brüser and Bogdan, 2017). Wnt signal stimulates several intra-cellular signal transduction cascades, including the canonical or Wnt/β-catenin dependent pathway and the non-canonical or β-catenin-independent pathway (Habas and Dawid, 2005). FGF pathway plays a role during metabolic disorders or in injured tissues, where it mediates metabolic functions, tissue repair, and regeneration, often by reactivating developmental signaling pathways. In another study using high resolution capillary electrophoresis and mass spectrometry methods, several changes in acute phase proteins including fibrinogen and haptoglobin, as well as lipoproteins, were identified in the plasma of an African penguin with confirmed aspergillosis (Valdivia et al., 2020).

In managed care scenarios, as are common to penguins, ideal testing should provide diagnostic impact as well as an option to use as a health surveillance tool to monitor the population for potential outbreaks so that early action may be taken. In addition, given the cost and labor involved in treatment, such tools should also provide prognostic value. As discussed here, it is doubtful that there will be a single tool that can provide such a broad implementation. Galactomannan and gliotoxin can aid in diagnosis but electrophoresis, hydroxybutyrate and antibody titers may best reflect prognosis. Overall, while the diagnostic role of new proteins and tools in penguins is unknown, their description broadens the perspectives of investigations and warrants additional studies to confirm their potential interest. In addition, various proteomic tools and methods may provide differing results; their use should be equally considered to aid in the potential identification of novel biomarkers.

## What are the diagnostic means in dolphins?

In marine mammals, aspergillosis is considered rare, but it has been recently reported with increasing frequency with more than two thirds of cases have been published after the year 2000 (Barley et al., 2007; Dagleish et al., 2008; Abdo et al., 2012). Cetaceans like dolphins live in aquatic media and *Aspergillus* spores are known to be also present in water. It is plausible that aspergillosis can occur in dolphins *via* water contamination although those in managed care may be further exposed due to environmental/air contamination in enclosed facilities. In dolphins, the risk factors are not clearly elucidated. A case report indicated a likely immune suppression related to a morbillivirus infection may have resulted in fatal aspergillosis in a free-ranging dolphin (Cassle et al., 2016). Overall, however, reports are not associated with severe neutropenia as seen in humans (Seyedmousavi et al., 2015), and instead, the development of aspergillosis is thought to be based on a chronic invasive process which is usually secondary to another condition like stress or other co-infection (Bunskoek et al., 2017), as it was seen in non-neutropenic humans with severe COVID-19 and/or influenza diseases. Underlying pulmonary disease may also affect host defense mechanisms in dolphins, leading to colonization and potential invasion of bronchial tissue by *Aspergillus* spp., generating one or several nodule(s) (Reidarson et al., 1998). In such a context, coughing, abnormal vocalizations, hard chuffing, radiological changes, and all signs related to tracheitis, bronchitis, pneumonia, pleurisy, can be reported. Other organs, like the brain, may also be infected following bloodstream dissemination (Dagleish et al., 2006; Dagleish et al., 2008; Abdo et al., 2012). *Aspergillus fumigatus* species is mostly involved in cetacean aspergillosis (Lamoth, 2016), followed by species belonging to the *Nigri* section and *Terrei* section, respectively (Balajee, 2009). For the curative treatment, several options exist, like nebulizing amphotericin B or some azole drugs directly into the blow hole (Bunskoek et al., 2017) or oral administration of voriconazole.

For the diagnosis of aspergillosis in dolphins, *antemortem* laboratory tools are largely less developed than for humans and not validated (Dagleish et al., 2008; Delaney et al., 2013; Cassle et al., 2016). Some diagnostic procedures are difficult to perform (Desoubeaux et al., 2014), especially because medical imaging (*e.g.* computed tomography (CT), magnetic resonance imaging (MRI), or even endoscopy) is not readily available. Positive culture from respiratory specimens may reflect a simple colonization of the upper airways or represent an environmental contaminant (Desoubeaux et al., 2014). Also, as reported in humans (Desoubeaux et al., 2014), blood cultures are usually also non diagnostic for aspergillosis in dolphins even in disseminated cases. Detection of galactomannan antigen is not sensitive: in a cohort of 87 common bottlenose dolphins (*Tursiops truncatus*), there were no differences between the *Aspergillus*-diseased cases *vs*. the controls (mean indices of 0.2 *vs*. 0.3) (Desoubeaux et al., 2018). Rare studies regarding the use of *Aspergillus* qPCR (usually targeting repeated regions of the ribosomal RNA subunits) in dolphins are available (Groch et al., 2018), so that it is difficult to definitively conclude on the diagnostic potential.

In contrast, as dolphins are not specifically immunocompromised during aspergillosis, serological testing to detect anti-*Aspergillus* antibodies is postulated as a reasonable option as reported in chronic infection in humans (Kurup, 2005; Persat, 2012). With a lab developed ELISA assay, antibody levels were higher in 32 diseased dolphins *vs.* 55 controls, at 1: 1024 *vs.* 1: 256 median titer dilution. The same study also reported the accuracy of a commercial western blot (WB) assay developed for use in humans that was adapted to dolphin serum *via* the use of a species specific conjugate antibody. Focusing on reactivity to four distinct WB bands that are also present in human samples at 30, 22, 18-20, and 16 kDa (Oliva et al., 2015), a minimum score of 5 (of a maximum of 16) was proposed to distinguish between the infected and non-infected dolphins (**Figure 2**). Seroconversion was consistently observed before positive fungal culture, and the specificity was 93%, regardless of the *Aspergillus* species involved. The cross-reactivity with other fungal genera was not observed and the WB testing also exhibited a valuable prognostic value.

To understand more about the pathophysiology of infection and possibly identify new tools, some studies have used mass spectrometry to study aspergillosis in dolphins (Desoubeaux et al., 2019). Several over-represented proteins which play a role in the adaptive immune response were identified, including MHC (major histocompatibility complex) proteins and others involved in catalytic activity like the NADPH-ubiquinone oxido-reductases or cytochrome b. The former are believed to be required for catalysis which functions in the transfer of electrons to the respiratory chain required for enzymatic activity to fight against aspergillosis. Noteworthy in the same study, no markers were shared with blood samples from infected humans, except one, so called Testis expressed 11 (Fragment) protein, >3-fold increased in both species and involved in the cell organization and biogenesis; metabolic process; regulation of biological process (Desoubeaux et al., 2018; Desoubeaux et al., 2019). Further studies are warranted to validate the relevancy of these proteins in the diagnostic process of aspergillosis.

## Conclusion

This brief overview highlights the difficulty in the diagnosis of aspergillosis in animals (Elad and Segal, 2018; Tell et al., 2019). *Antemortem* lab-based methods derived from human medicine need to be implemented and validated with the goal to increase the accuracy of the diagnosis in animals. Proteomic studies may reveal new biomarkers which may be unique to infection in some animal species or possibly aid in enhanced diagnosis in mammals including humans. When one can do without invasive specimens and advanced imaging, using blood for such investigations represents great opportunity to commence a pathway to diagnosis. However, results from these studies need to be carefully interpreted (Desoubeaux et al., 2018; Desoubeaux et al., 2019). Animals may more likely to be colonized and/or chronically infected than humans. Moreover, the clinical signs depend on the host species and the underlying conditions or comorbidities may greatly vary. In addition, the lack of consensus regarding the disease classification in animals can have great impact on the reliability of all the studies published thus far (Donnelly et al., 2020), and that protein libraries available are lacking data related to non-traditional species *vs*. humans and laboratory animals (Mi et al., 2017). Lastly, in the absence of neutropenia and severe immune compromise as seen in transplant recipients, the pathology and immune response of aspergillosis in birds and dolphins should be rather compared to those of subacute/chronic human aspergillosis and any diagnostic tools which are developed and/or adapted address these opportunities for sensitive and specific options for antemortem diagnosis.

## Data availability statement

The original contributions presented in the study are included in the article/supplementary material. Further inquiries can be directed to the corresponding author.

## Author contributions

GD led and supervised the writing, CC edited the text (English native speaker) and brought specific comments, AC corrected the manuscript and proposed some improvements. All authors contributed to the article and approved the submitted version.

## Conflict of interest

GD was specifically invited by the editorial board to write this manuscript.

The remaining authors declare that the research was conducted in the absence of any commercial or financial relationships that could be construed as a potential conflict of interest.

## Publisher’s note

All claims expressed in this article are solely those of the authors and do not necessarily represent those of their affiliated organizations, or those of the publisher, the editors and the reviewers. Any product that may be evaluated in this article, or claim that may be made by its manufacturer, is not guaranteed or endorsed by the publisher.
